# Determining research knowledge infrastructure for healthcare systems: a qualitative study

**DOI:** 10.1186/1748-5908-6-60

**Published:** 2011-06-06

**Authors:** Moriah E Ellen, John N Lavis, Mathieu Ouimet, Jeremy Grimshaw, Pierre-Olivier Bédard

**Affiliations:** 1 Centre for Health Economics and Policy Analysis, McMaster University, Hamilton, Ontario, Canada; 2 Department of Clinical Epidemiology and Biostatistics, McMaster University, Hamilton, Ontario, Canada; 3 Department of Political Science, McMaster University. Hamilton, Ontario, Canada; 4 McMaster Health Forum, McMaster University. Hamilton, Ontario, Canada; 5 Department of Political Science, Université Laval, Quebec, Quebec City, Canada; 6 Ottawa Health Research Institute, Ottawa, Ontario, Canada

## Abstract

**Background:**

This study examines research knowledge infrastructures (RKIs) found in health systems. An RKI is defined as any instrument (*i.e*., programs, interventions, tools) implemented in order to facilitate access, dissemination, exchange, and/or use of evidence in healthcare organisations. Based on an environmental scan (17 key informant interviews) and scoping review (26 studies), we found support for a framework that we developed that outlines components that a health system can have in its RKI. The broad domains are climate for research use, research production, activities used to link research to action, and evaluation.

The objective of the current study is to profile the RKI of three types of health system organisations--regional health authorities, primary care practices, and hospitals--in two Canadian provinces to determine the current mix of components these organisations have in their RKI, their experience with these components, and their views about future RKI initiatives.

**Methods:**

This study will include semistructured telephone interviews with a purposive sample region of a senior management team member, library/resource centre manager, and a 'knowledge broker' in three regional health authorities, five or six purposively sampled hospitals, and five or six primary care practices in Ontario and Quebec, for a maximum of 71 interviewees. The interviews will explore (a) which RKI components have proven helpful, (b) barriers and facilitators in implementing RKI components, and (c) views about next steps in further development of RKIs.

**Discussion:**

This is the first qualitative examination of potential RKI efforts that can increase the use of research evidence in health system decision making. We anticipate being able to identify broadly applicable insights about important next steps in building effective RKIs. Some of the identified RKI components may increase the use of research evidence by decision makers, which may then lead to more informed decisions.

## Background

Significant worldwide investments that are made in biomedical and health research are underutilised because of challenges to knowledge translation (KT). KT is defined as 'the exchange, synthesis and ethically-sound application of researcher findings within a complex system of relationships among researchers and knowledge users to improve health' [1]. Evidence shows that health systems frequently fail to optimally use research evidence, which leads to inefficiencies, reduced quantity and quality of life for citizens, and lost productivity [2].

Multiple factors determine the nature and extent of engagement in KT by different stakeholder groups. A common challenge that all decision makers face relates to the lack of knowledge management skills and infrastructure (*i.e*., the sheer volume of research evidence currently produced; access to research evidence; time to read; and skills to appraise, understand, and apply research evidence). Better knowledge management is necessary but probably insufficient to ensure effective KT given other challenges that may operate at different levels, including the healthcare system (*e.g*., financial disincentives), healthcare organisation (*e.g*., inappropriate skill mix, lack of facilities or equipment), healthcare teams (*e.g*., local standards of care not in line with desired practice), individual professionals (*e.g*., knowledge, attitudes, and skills), and patients (*e.g*., low adherence to medical advice) [3]. Frequently, multiple challenges are present at different levels of the healthcare system. A further challenge for KT relates to the dynamism and constant evolution of healthcare. As a result, KT approaches and activities need to recognise and keep pace with the changes in the healthcare sector.

Physical and cognitive access to evidence are challenges to KT for health system managers and policy makers. Three primary KT approaches target these groups: push, pull, and exchange efforts [4]. The *push *approach includes activities undertaken by researchers to package and disseminate research evidence outside the scholarly community. *Pull *activities focus on the efforts by health system managers and policy makers to access and use research evidence. *Exchange *activities focus on building and maintaining relationships between researchers and managers and policy makers. We propose to study research knowledge infrastructures (RKIs) that build on push, pull, and/or exchange components.

In this study, RKI is defined as any instrument (*i.e*., programs, interventions, tools, devices) implemented in key organisations and broader health systems of which they are a part (hereafter called health systems) in order to facilitate access, dissemination, exchange, and/or use of evidence. These instruments can be subdivided into two broad categories: (1) technological instruments (*e.g*., electronic databases, search engine) and (2) organisational instruments (*e.g*., documentation specialists; data analysts, such as epidemiologists; knowledge brokers who, for example, manage the collaboration between the organisation and external information and knowledge producers; and training programs to help in, for example, searching for information, appraising information, adapting and using both statistical data and academic research) [5]. Multiple types of evidence can be used by health system managers and policy makers, including (among others) administrative information, legal information, academic research, financial data, and surveillance data. In this project, we will be focusing on access to and utilisation of the following types of evidence: academic research outputs (*i.e*., articles, research reports, and books) and population and health system data (*i.e*., surveillance data, service utilisation data, and other nonfinancial performance data).

Findings from largely observational studies suggest that a number of factors are associated with the use of research evidence by policy makers, including personal contact with researchers, timeliness and relevance of research, and inclusion of summaries with recommendations [6,7]. These and other findings have led to the development of a number of KT approaches targeting policy makers and senior health system managers[8,9]. Most of these approaches have a strong theoretical basis and face validity. We developed an RKI framework that identified potential RKI components that a healthcare system or a hospital could have in its RKI. The framework includes the broad domains of Climate for Research Use, Research Production, Activities Used to Link Research to Action, and Evaluation. Table 1 includes the detailed elements within each RKI domain. We conducted an environmental scan and scoping review to conduct a preliminary assessment of possible RKI components that exist in various countries and in the applicable literature.

**Table 1:** Possible organisational level components of a research knowledge infrastructure (RKI)

Domain	Elements
I. Climate for Research Use	• Accreditation acknowledges and rates the use of research evidence in decision making
	• Mission, vision, values, and strategic plan reflect the value placed on the use of research evidence in decision making
	• Structures or positions exist within the organisations to ensure accountability for using research evidence in decision making
	• Clear points of contacts within organisations regarding where to turn to obtain research evidence
	• Formal and informal relationships with people outside the organisation who can assist in obtaining the appropriate research evidence
	• Recruitment and retention strategies that reflect the value of the use of research evidence in decision making
	• Recognition of employees who use research evidence within the organisation

II. Research Production	• Participating in regular priority setting processes for the research evidence needed to meet managerial and policy-making needs
	• Ensuring that the appropriate research commissioning capacity is in place to commission or execute the research if it is deemed high priority yet no applicable research is available

III. Activities Used to Link Research to Action (4 parts)	

IIIa. Push Efforts	• Knowledge intelligence service that scans the literature and distributes relevant research evidence throughout the organisation
	• Individual(s) responsible for identifying teaching moments to profile research evidence

IIIb. Facilitating Pull Efforts	• Enabling 'easy access' to research evidence through physical tools (*e.g*., information technology systems)
	• Enabling 'easy access' to research evidence through appropriate resources (*e.g*., websites with optimally packaged reviews)

IIIc. Pull Efforts	• Summarising or conducting primary research via a rapid response unit that supports the use of research in decision making
	• Decision-making processes that promote the use of research in decision making
	• Self-assessment tools that focus on capacity to find and use research evidence in decision making
	• Training and continuing education that focus on finding and using research evidence in decision making
	• Use of dedicated staff to 'pull' research into decision making

IIId. Exchange Efforts	• Regular meetings that highlight relevant research

	• Interactive workshops that focus on the use of research in decision making
IV. Evaluation of Efforts	• Monitoring and evaluation efforts on the use of research in decision making

We conducted an environmental scan of current RKIs in two Canadian provinces (*i.e*., extraction of key information from organisations' websites and informal interviews with key informants). Our informal interviews with 17 key informants were aimed at generating examples of tools, interventions, and components of RKIs. With respect to the RKI domain of Climate for Research Use, many of the informants referred to their organisation's strategic plan, which explicitly supported the use of research evidence in decision making. Most of the informants reported having existing relationships with researchers, and formal relationships were often facilitated by the structure of the organisation. In the RKI domain of Research Production, some informants suggested the existence of priority-setting processes, and although research production appears limited within most organisations, some suggested they have the capacity to commission research. Efforts to disseminate research findings both within and across organisations and the usage of knowledge brokers were mentioned when discussing push activities.

Many of the key informants mentioned that the categories within the Facilitating Pull domain enabled them to access research evidence to be used in decision making (*i.e*., organisations benefited from full access to bibliographic databases through partnerships, some organisations have comprehensive library services, and some have information tools to assist management). Within the Pull Activities domain, two key informants identified the establishment of rapid response units that produced information syntheses within approximately two weeks for managers, decision makers, and policy makers. Furthermore, various forms of training programs were discussed that educated participants in the use of research evidence in decision making. No examples of the Evaluation of Efforts to Link Research to Action were provided. The environmental scan demonstrates that health system managers and policy makers in Ontario and Quebec have instituted in their organisations and in the health system as a whole varying components of an RKI; yet, the breadth, depth, usage, and effectiveness of these efforts have yet to be determined.

We also conducted a scoping review as a preliminary assessment of the size and scope of the available literature on the effectiveness of particular models, or specific components, of RKI, as well as regarding the problems they are addressing, other features of RKIs that may influence their design, and related implementation considerations. We manually reviewed (a) http://healthsystemsevidence.org, a continuously updated repository of syntheses of research evidence about governance, financial, and delivery arrangements within health systems and about implementation strategies that can support change in health systems; (b) a database of studies used in a systematic review of the factors that influence the use of research evidence in public policy making; and (c) other studies or reports that we were aware of that were applicable to our scoping review. In reviewing these databases, we were trying to identify all primary studies that address RKI either in whole or in part.

We were unable to identify any studies evaluating the effects of a full RKI on the use of evidence by health system managers and policy makers; however, the scoping review did uncover 25 qualitative studies and one randomised control trial that addressed different components of the RKI framework (for a list of included studies, please contact MEE). The majority of the studies (25 studies) addressed categories within the Climate for Research Use domain, while the next most addressed domains were Activities Used to Link Research to Action, Facilitating Pull, and Pull Activities (16 studies). Fourteen studies evaluated the Research Production domain, and 10 studies addressed exchange activities. A minority of the studies (seven studies) reported on Push Activities, and even fewer (two studies) reported on the Evaluation of Efforts to Link Research to Action. The one subdomain that was reported most often, by 20 studies, as influencing the use of research in decision making was the formal and informal relationships to people outside the organisation who can assist in obtaining the appropriate research evidence (see Additional File 1 for illustrative examples associated with each category). This scoping review illustrates that while numerous independent initiatives are in place in different healthcare systems, no one RKI framework has been developed or studied to determine overall effectiveness.

Building on the environmental scan and scoping review, we propose to conduct interviews with health system managers and policy makers in Ontario and Quebec to determine which RKI components have actually been implemented and are perceived to have added value in their organisations, the barriers and facilitators to implementing these RKI components, and views about how to further support RKI development. The RKI project will be a springboard to cross-organisation and cross-system research to better understand how to match particular RKI elements to different contexts.

## Objectives

The objectives of our study are as follows:

• To profile the RKI of three key types of healthcare organisations in two Canadian provinces (Ontario and Quebec): regional health authorities, primary care practices, and hospitals

• To identify barriers and facilitators to implementing various RKI components

• To determine what these organisations view as important next steps in building effective RKIs

## Methods

We will conduct in-depth, semistructured telephone interviews with a senior management team member (who will be focused more on organisational infrastructure), library/resource centre manager (who will be focused more on technical infrastructure), and (for some types of large organisations) a knowledge broker. These interviews will be conducted in three regional health authorities (three participants interviewed in each, for a total of 18 interviews), five or six purposively sampled hospitals (depending on the hospital's size, we will interview two or three participants in each hospital, for a total of 29 interviews), and five or six primary care practices (with two individuals interviewed in each organisation, for a total of 10 to 12 interviews) in Ontario and Quebec.

We are interested in examining organisations that have already participated in strategic behaviour with respect to RKI (or that are linked to such organisations). We identified a sample of these organisations by examining the publicly available list of participants that have been a part of the Canadian Health Services Research Foundation's Executive Training for Research Application program. This program is geared towards health system managers and policy makers in Canadian health system organisations and aims to develop capacity and leadership to optimise the use of research evidence. The organisations from which these participants are drawn committed to having one or more of their senior executives enroll in the program and participate in four 1- to 2-week training sessions over a two-year period, supporting their executive(s) in designing and executing an evidence-based 'intervention project', and sending their Chief Executive Officer to a final meeting where the intervention project would be discussed. We augmented the sample based on our knowledge of other organisations that have demonstrated strategic behaviour with respect to RKI.

The focus of the interviews will be to explore which RKI components have been implemented and are perceived to have added value in the participant's organisation (and which not), the barriers and facilitators encountered in implementing these RKI components, and views about next steps in supporting the further development of RKIs. Semistructured interviews were utilised because they allow the interview participants to respond freely and focus on the area of the framework that the respondents think most apply to their organisation [10]. Furthermore, using semistructured interviews permits the interviewer to probe issues that may be of interest to the current research but that are not specifically addressed by the interview guide [11].

We will tape and transcribe all interviews, and they will be verified by the interviewer before analysis begins. NVivo, Version 9, (QSR International, Cambridge, MA) will be used for data management. A constant comparative method for the thematic analysis of the interview data will be used (with one very experienced qualitative researcher working with one doctoral student to identify themes). Interviews will be analysed in clusters based on their organisations, meaning all interviews from one organisation will be analysed in tandem, followed by all other institutions within that organisational context (*i.e*., regional health authorities, hospitals, and primary care practices). First we will read the entire interview to get a sense of the whole interview and initial impressions. Then we will read the text a second time, code units of text, and compare initial codes. Coded segments will then be reanalysed, coded into subcategories, and compared again. Two researchers will constantly compare the coding during each stage.

## Discussion

Upon completion of the qualitative study, we will produce a report that describes the RKI status in regional health authorities, hospitals, and primary care practices; identifies barriers and facilitators to implementing various RKI components; and provides an overview of what these health system managers and policy makers view as important next steps in building effective RKIs. Based on these qualitative findings, we will develop a protocol for a cross-sectional survey that will generate more generalisable information.

The environmental scan and scoping review have identified key elements of RKI, and this qualitative work will explore the extent of the use of these elements in different settings. We anticipate being able to identify broadly applicable insights about important next steps in building effective RKIs. This is the first qualitative examination of potential RKI efforts that can increase the use of research evidence in health system decision making. Some of the identified RKI components may increase the use of research evidence by decision makers, which may then lead to more informed decisions, and hopefully to a strengthened health system and improved health.

## Competing interests

The authors declare that they have no competing interests.

## Authors' contributions

MEE coordinated the study, conducted the scoping review, and drafted the manuscript. JNL conceived and designed the study, oversaw the scientific direction, and helped to draft the manuscript. MO contributed to the conception and design and conducted the environmental scan. JG contributed to the conception and design of the study. POB conducted the environmental scan. All authors read and approved the final manuscript.

## Supplementary Material

Additional file 1 **Illustrative examples of quotes from the scoping review associated with the research knowledge infrastructure categories**. A scoping review was conducted looking at all relevant literature to determine if the research knowledge infrastructure (RKI) framework that we developed was supported by the empirical literature. This additional material contains excerpts from the scoping review that demonstrate quotes associated with the appropriate RKI categories [12-25]. Click here for file
