# Supplementary material for: Determining research knowledge infrastructure for healthcare systems: a qualitative study
Source: Implement Sci. 2011 Jun 6;6:60. doi: 10.1186/1748-5908-6-60 (PMC3123231; doi:10.1186/1748-5908-6-60)
Supplement: Additional file 1 — Illustrative examples of quotes from the scoping review associated with the research knowledge infrastructure categories. A scoping review was conducted looking at all relevant literature to determine if the research knowledge infrastructure (RKI) framework that we developed was supported by the empirical literature. This additional material contains excerpts from the scoping review that demonstrate quotes associated with the appropriate RKI categories [12-25]. [file 1748-5908-6-60-S1.DOC]

**Illustrative examples of quotes from the scoping review associated with the research knowledge infrastructure categories**

| **RKI categories and elements (with the # of studies addressing each)** | **Illustrative quotations from studies** |
| --- | --- |
| **Climate for research use (25)** |  |
| Accreditation acknowledges and rates the use of research evidence in decision making (0) |  |
| Mission, vision, values, and strategic plan reflect the value of the use of research evidence in decision making (13) | "Respondents consistently emphasized the importance of evidence-based practice, and many reflected positively on the government commitment to both its adoption and development of EBHPR [Evidence Based Health-Promotion Resources] resources" [12, p.256] |
| Structures or positions accountable for encouraging research use in decision making (14) | "our interviews in the Ministry of Transport indicated that their planning department was recognized within as a channel for new knowledge, although staff members in planning departments are not aware of their roles as intermediaries" [13, p.38]. "Planning departments are the primary organizational means of diffusion of research in the Norwegian ministries. …The departments were designed to conduct some research themselves and act as liaisons between the research community and users in and out of the ministries" [13, p.44] |
| Clear points of contacts within the organization regarding where to turn to obtain research evidence (6) | "One department had developed guidelines to support their staff to collaborate effectively with their economics unit…" [14, p.4] |
| Formal and informal relationships to people outside the organization who can assist in obtaining the appropriate research evidence (20) | "The most frequently nominated strategy for improving the use of research in respondents' organisations was improved access to research and researchers (32%). Participants' suggestions included: "building bridging systems between researchers and policy makers" and "standing arrangement with key research groups and key research people who can readily assist in policy making" [15, p.7]. |
| Recruitment and retention/ HR strategies that reflect the value of the use of research evidence in decision making (4) | " Many position descriptions in health require a qualification that includes some introduction to economics or health economics e.g. Masters of Public Health or Masters of Health Administration" [14, p.4]. |
| Recognition of the individuals who use research within the organization (1) | Implicit: One of the advantages with respect to the Sheps centre "such programs may also be necessary to circumvent the university' incentive structures and create incentives and rewards that are supportive of state policy work" [16, p.147]. |
| **Research production (14)** |  |
| Regular priority setting processes that link research to action (10) | Lack of: "The most commonly cited reason attributed to the limited usability of existing data was that policymakers’ needs do not drive research… academic researchers generally follow their own interests when choosing what studies to conduct or tailor them to specific requests for grants. Similarly, the synthesis of existing research in the form of systematic reviews is driven by the researchers’ particular interests" [17, p.189].  "Establish strong links with policymakers and involve stakeholders in the work: This advice was reinforced by: 1) the (quantitative) survey finding that a high proportion (88%) of GSUs [Government Support Units] involved target users in the selection of topics or the services undertaken" [18, p.3]. |
| Research commissioning capacity (7) | "Mechanisms used to secure this advice varied among the departments of health...using a mixture of in-house and external sources... The range of options seen in practice extended from sourcing services from external groups…" [14, p.3]. |
| **Activities used to link research to action: Push activities (7)** |  |
| Knowledge intelligence service that scans the literature and highlights and distributes relevant knowledge.(7) | "The moderately interactive KTE [knowledge translation and exchange] intervention included tailored, targeted messages plus access to health-evidence.ca” [19, p.5]. "The results indicate that the 'right' evidence, 'pushed' out to the right decision maker working in an organization supportive of evidence-informed decision making, leads to outcomes in the hypothesized direction" [19, p.10]. |
| One or more individuals within the organization are responsible for identifying teaching moments (i.e. windows of opportunity) to profile research (0) |  |
| **Activities used to link research to action: Activities to facilitate pull (16)** |  |
| Enabling "easy access" to the appropriate research: Physical tools to enable access (5) | "Data were collected and held within multiple sources with some outside NHS [National Health Service] boundaries (e.g. private sector), rendering it difficult or impossible for network teams to access" [20, p.215]. |
| Enabling "easy access" to the appropriate research: Content/ resources (14) | "Structured databases like the NHS EED [National Health Service Economic Evaluation Database] do fulfill a useful function in that they help potential users quickly identify relevant studies and provide comments on their quality and relevance…" [21, p.78]. |
| **Activities used to link research to action: Pull activities (16)** |  |
| Summarizing or conducting primary research that facilitates the use of research in decision making (7) | "Infarmed in Portugal (for certain new drugs) and National Institute of Clinical Excellence (NICE) in the United Kingdom (for health technologies having a major impact on the health-care system) have led to an increase in the formal use of economic evaluation. However, it should be noted that these institutional changes themselves are quite recent” [22, p.11]. |
| Decision making processes that promote the use of research in decision making (10) | "A lack of understanding of evidence- informed decision-making and the need for more education of and communication with policymakers was also noted" [23, p.5]. |
| Self assessment tools that highlight the importance of research use in decision making (1) | "Two main approaches that have been described are the use of tools to assess organizational capacity to acquire and apply research evidence and continuing education program” [15, p.9]. |
| Training and continuing education that include research use in decision making (12) | "Apparently, the decision makers are of the view that a sound training in health economics could contribute to a wider use of health economic studies" [24, p.191]. |
| Use of dedicated staff to 'pull' research into decision making (7) | “This role fell to the information and Data Lead or the network Lead Manager who supported the rest of the network team and wider network partners in their use of data. Information Leads effectively turned data into information for the rest of the network team and the network” [20, p.216]. |
| **Activities used to link research to action: Exchange activities (10)** |  |
| Regular meetings that highlight relevant research (6) | "Among the array of services undertaken in response to requests from public policymakers, GSUs [government support units] are most likely to convene expert meetings to discuss available research (82%) and undertake short-term research projects (79%)" [25, p.4]. |
| Interactive workshops that focus on the use of research in decision making (6) | "Practitioners were very supportive of workshops when they were conducted to introduce a new resource.… it was clear that practitioners preferred active dissemination in addition to passive forms of dissemination" [12, p.257]. |
| **Evaluation of efforts to link research to action (2)** |  |
| Implementation and evaluation efforts on the use of research in decision making (2) | "Between one-half and two-thirds of organizations do not collect data systematically about uptake, and roughly the same proportions do not systematically evaluate their usefulness or impact in other ways" [25, p.12]. |
